# Outstanding Contributions to Aerosol Pulmonary Drug Delivery

**DOI:** 10.14356/kona.2026011

**Published:** 2025-06-21

**Authors:** Anthony J. Hickey, Erik S. Pena, Sara E. Maloney Norcross

**Affiliations:** 1 Technology Advancement and Commercialization, RTI International, USA; 2 Division of Pharmacoengineering and Molecular Pharmaceutics, Eshelman School of Pharmacy, University of North Carolina at Chapel Hill, USA

**Keywords:** dry powder inhaler, pulmonary, manufacturing, physicochemical characterization, aerosol dispersion

## Abstract

Dose accuracy and precision for pulmonary drug delivery have been core elements of therapy for asthma for 70 years. As the technology has developed, its application has spread to various diseases. For many inhaled products, solid-state chemistry, the nature of the drug particles, and their relationship to other particles in the formulation underpin success in disease treatment. Methods of manufacturing yield unique particle systems whose properties support the range of doses required to treat diseases with low- and high-potency drugs requiring high and low doses, respectively. To ensure the quality of these particulate products, which correlates with safety and efficacy, comprehensive characterization of their physicochemical properties and aerosol performance is required. The delivered dose and aerodynamic particle size distribution are key characteristics related to lung exposure required in clinical efficacy trials for non-communicable, genetic, environmental, and communicable (i.e., infectious) diseases. The breadth of inhaled therapy has increased significantly since the introduction of the initial products in the last century. The desire to treat genetic diseases, such as cystic fibrosis, and the emergence of new approaches to lung therapy during the COVID-19 pandemic are opening up new opportunities in inhaled biologicals that are anticipated to lead to future developments.

## Graphical Abstract

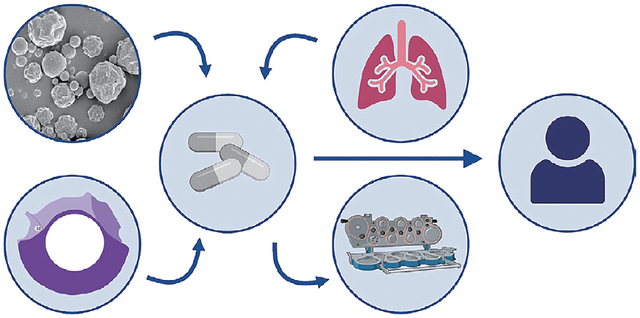

## 1. Introduction

Therapeutic aerosols have been used for most of recorded time. Particulates have been an essential part of many of these treatments and involved the use of smoke generated by the combustion of plants containing pharmacological agents, such as stramonium alkaloids (Sanders, 2011). Modern inhaled aerosols may be considered to have originated when the technological ability to create airborne particulates at accurate and precise doses coincided with the emergence of instruments and techniques sensitive enough to analyze such materials. These developments allowed both control and monitoring of the quality of products to ensure that safe and effective doses were delivered reproducibly.

Pharmaceutical inhalation aerosol technology combines drug formulation, a metering system, and a device for effective dose delivery in sizes that are known to deposit in the lungs (Hickey, 2016). Particles can be delivered using each of the primary categories of inhalers, including nebulizers, metered dose inhalers (MDIs), and dry powder inhalers (DPIs). Nebulizers deliver aqueous formulations that may contain suspended particles, such as budesonide, which are used for treating pediatric asthma (Szefler, 1999). Pressurized MDIs are non-aqueous systems from which aerosols are generated under the influence of liquefied propellants. Particles may be suspended in the propellant, where the presence of engineered particles is critical for performance, as is the case in Bevespi Aerosphere^®^ (AstraZeneca) (Ferguson et al., 2018). However, this paper will focus on DPIs, including the variety of methods employed to manufacture drug particles and the strategy utilized to deliver a defined dose over the range required to treat a variety of pulmonary diseases.

## 2. Manufacturing

**Fig. 1:**
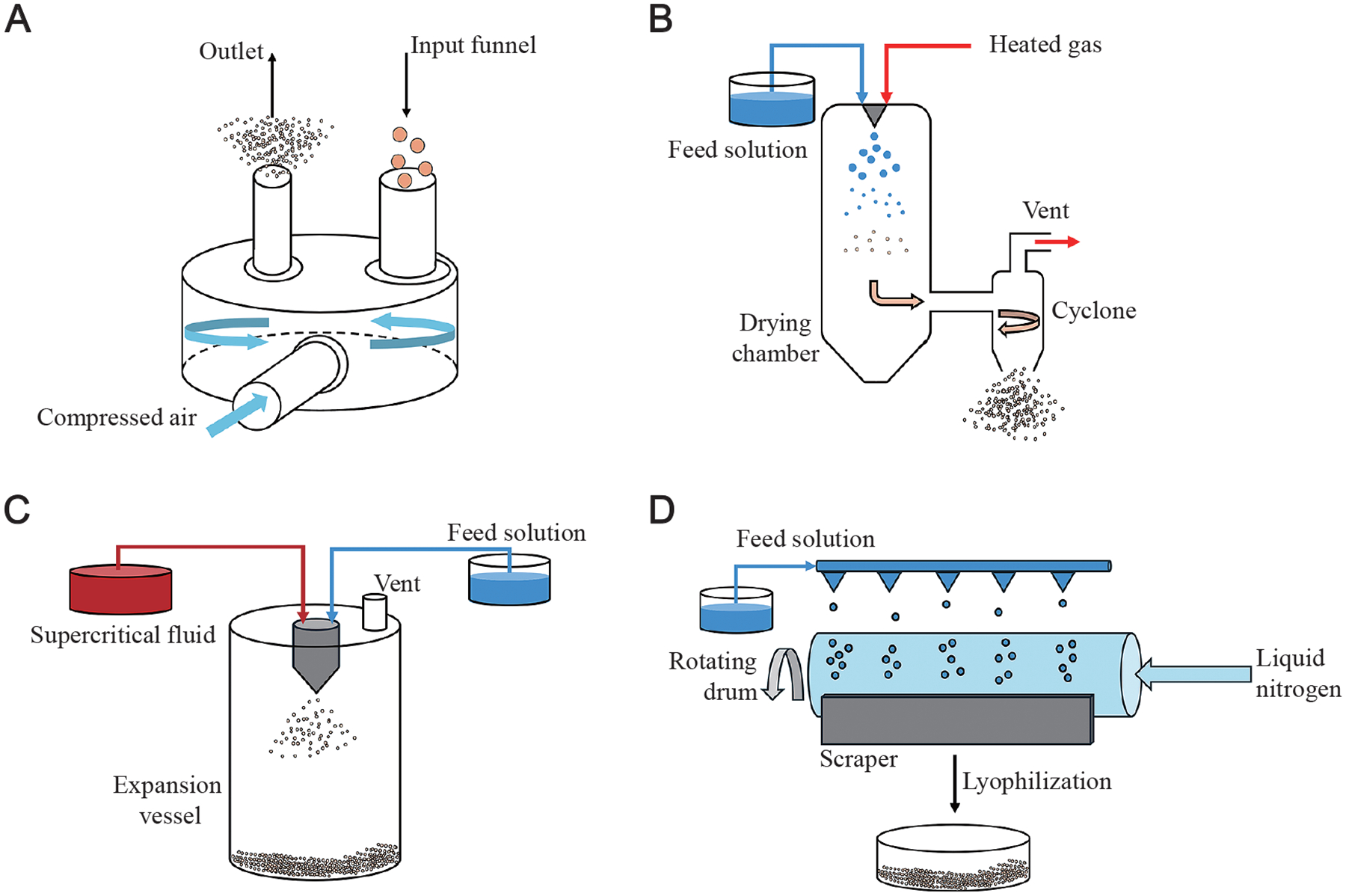
Common manufacturing methods of inhalable dry powders: **(A)** jet milling, **(B)** spray drying, **(C)** supercritical fluid, and **(D)** thin film freezing.

Several manufacturing methods have been explored to produce drug particles for lung delivery via dry powder inhalation (Fig. 1). With their own sets of advantages and disadvantages, these formulation processes can have significant effects on the physicochemical properties, aerodynamic behavior, and therapeutic efficacy of particles. By modifying the parameters during manufacturing, one can engineer and tune the properties of a dry powder to meet their desired characteristics. Particle engineering has been explored through the years with different manufacturing methods, and this section highlights the commonly used methods in the field with their process operation.

### 2.1 Milling

One of the most common methods used to produce inhalable dry powders is micronization by jet milling (Fig. 1(A)) (Hickey, 2018a; Shetty et al., 2020; Yang et al., 2024). In this process, compressed air or gas is fed into a chamber with an active pharmaceutical ingredient (API), and the high velocity of the particles causes them to impact with one another and against the walls of the apparatus. The mechanical energy generated by particle–particle and particle–wall interactions, including impact and shear forces, induces fracturing, reducing particle size. There are different variations in the jet milling process (e.g., fluidized-bed, oval-chamber, reverse jet), and the advantage of this method is that it is convenient and cost-effective. However, particles generated by this process can have inconsistent shapes and solid-state characteristics, formation of surface amorphous regions, and cohesion of particles to other particles and the walls. Co-milling and wet milling have been explored as ways to minimize particle cohesion. Furthermore, milled particles are often blended with a coarse lactose carrier to improve particle dispersion and enable filling and metering from DPIs. Milling is currently the primary process by which FDA-approved dry powders for inhalation are manufactured.

### 2.2 Spray drying and spray freeze drying

Since the 1980s, spray drying has been explored as an alternative to jet milling and is now a commonly used method to produce consistent inhalable dry powders in research (Fig. 1(B)) (Hickey, 2018a; Shetty et al., 2020; Vehring, 2008; Yang et al., 2024). The process involves pumping a feed solution containing API into a spray nozzle, where it comes into contact with heated air or nitrogen gas to atomize the solution into droplets. The elevated temperature of the gas begins to dry the droplets in the drying chamber, generating dried particles. The particles are then separated from the drying gas in a cyclone and deposited in a collection vessel. Spray drying can create amorphous particles, which are desirable for dry powder inhalation because they dissolve more rapidly and have greater bioavailability than their crystalline counterparts; however, the amorphous nature of the powder may affect its stability during storage due to moisture uptake (Wu et al., 2010). This method is a one-step, continuous process that provides unique particle engineering approaches based on process parameters and added excipients. Optimization is possible and may be necessary due to the elevated temperatures and shearing of the heated gas, which may lead to API degradation in sensitive samples.

Spray freeze drying offers an alternative to spray drying for heat-sensitive drugs and biologicals. Here, a feed solution is sprayed from a nozzle that is met with compressed air or nitrogen gas that is not heated (Farinha et al., 2023; Rostamnezhad et al., 2022). The droplet solution is sprayed into liquid nitrogen, where it will rapidly freeze and solidify. After the spraying process, the liquid nitrogen and moisture in the solidified droplets are dried or removed under vacuum. One disadvantage of this process compared to conventional spray drying is that it is now a two-step process, which can make scale-up processes more complex. Overall, spray drying has offered researchers a new method to tune the solid-state characteristics of dry powders by optimizing their process parameters, with emerging technologies making their way into pharmaceutical development (Baumann et al., 2021).

### 2.3 Supercritical fluid

Supercritical fluid (SCF) is a more recent method for preparing particles for inhalable drug delivery systems. There are several different process methods in SCF technology; however, the most efficient method is the rapid expansion of supercritical solutions process (Fig. 1(C)) (Kankala et al., 2017). With this technique, powders are formed by first dissolving the API in a supercritical fluid solvent (e.g., ethanol or carbon dioxide) and mechanically precipitating the solids by rapidly expanding the solution. The process conditions are mild and suitable for sensitive APIs. Additionally, carbon dioxide is often used in this process due to being non-toxic, sustainable, and environmentally friendly. SCF offers tunability in particle formulation and engineering and has been adapted preclinically to produce fine particles with desirable size distributions for lung deposition. However, this process is still in its infancy stage, and scaling up is currently being investigated, with no product available on the market.

### 2.4 Thin film freezing

Microparticles of low density with desirable porosity and size can be formulated using thin film freezing (TFF) (Fig. 1(D)) (Hufnagel et al., 2022; Pardeshi et al., 2022). First, a solution containing the API is sprayed onto a metal plate or drum that is cooled to a set temperature in order to freeze the sprayed droplets. Once frozen, the film is scraped and collected for further processing via milling or solvent evaporation to obtain particles. The rate at which the droplet freezes can strongly affect the particle structure. This process can be applied to sensitive APIs to produce powders of a desirable size with high yield. The primary limitations of TFF are the process stabilization and reduced particle surface area to volume ratio. This technology is in its late stages of translation to the clinic, with two formulations reaching Phase II clinical trials, namely voriconazole inhalation powder and tacrolimus inhalation powder created by the former company TFF Pharmaceuticals (TFF Pharmaceuticals Inc., 2024).

### 2.5 Emulsion/solvent evaporation

Polymers have also been explored to encapsulate APIs within particles as a drug delivery system for inhalation (Hoppentocht et al., 2014; Maloney Norcross et al., 2024). A commonly used method to develop particles with high internal porosity at the micron- and nano-scales is emulsion/solvent evaporation techniques. For example, in the double-emulsion process, a polymer and drug are dissolved in a solution containing both hydrophobic and hydrophilic solvents. The solution is emulsified using a homogenizer or sonicator, in which hydrophilic emulsions are dispersed through a hydrophobic solvent. Water with a dissolved surfactant is then added, and the solution is emulsified again to create a water–oil–water emulsion, in which the polymer encapsulates the drug. The solution is then dried, leaving a dry powder. This process has the advantage of encapsulating both hydrophobic and hydrophilic APIs. However, this is not a single-step or continuous process, making scaling up complicated. Poly(lactic-co-glycolic acid) (PLGA) is a common polymer used to encapsulate APIs via emulsion due to its pH sensitivity and biodegradable nature. However, this formulation method has yet to have an approved commercial product for inhaled delivery. The most notable approved drugs for emulsion microparticles are administered via intramuscular injection (e.g., Lupron Depot^®^).

## 3. Dose considerations

The potency of the drug, and by inference, the required dose to be delivered, informs the selection of the manufacturing method. High-potency drugs, such as bronchodilators and corticosteroids, may be dosed in as little as microgram quantities. These drugs are frequently manufactured using jet milling techniques, followed by lactose blending for efficient aerosolization. The use of a coarse lactose carrier particle is beneficial in that it can aid in overcoming the cohesive forces between micronized particles, enhancing flow and preventing aggregation, ultimately improving aerodynamic performance (Mahar et al., 2022). Furthermore, blending with a carrier particle increases the total mass of the dose, allowing for more reproducible filling of drugs into dose metering systems (Maloney Norcross et al., 2024). Although lactose is the most common carrier particle in commercial DPIs, other sugars, such as mannitol, trehalose, and erythritol, have been examined in the literature as carriers (Mahar et al., 2022; Rahimpour et al., 2014).

Low potency drug formulations (e.g., antibiotics) typically cannot include large carrier particles because the powder dose required to achieve therapeutic concentrations of the API within the lungs would be impractical. As such, alternative techniques have been employed to achieve high-dose delivery. While the definition of “high-dose” delivery varies from source to source, it is generally agreed upon that high-dose delivery involves tens to hundreds of milligrams, a dose that is required by many antibiotics for pulmonary delivery (Hickey, 2024; Hofmann et al., 2024; Smyth, 2024; Son et al., 2021). Spray drying and spray freeze drying are frequently employed techniques to achieve low-density, amorphous drug microparticles. Spray-dried and spray freeze-dried particles often exhibit lower interparticulate forces than those observed with jet-milled particles, facilitating sufficient particle deaggregation without the need for a lactose carrier particle, which in turn increases the drug content in the powder (Maloney et al., 2023b). Excipients may be required for a variety of reasons, including stability and aerodynamic performance enhancements. However, adding excipients decreases the drug content in the powder, highlighting that careful considerations must be made to balance both the need for the excipient’s conferred properties and the feasibility of delivering the total powder dose (Hickey, 2014, 2024). Alternatively, researchers have reported excipient-free antibiotic dry powder formulations that employ the controlled aggregation of jet-milled drug microparticles (Brunaugh et al., 2017). This method eliminates the need for added excipients and maximizes drug content in the inhalation powder.

## 4. Characterization

Analyzing the physicochemical properties of a dry powder is a necessity to ensure that the desired drug potency and delivery characteristics are met. Many aspects of the physicochemical properties can affect the performance of the final product. For example, the crystallinity state of a dry powder can influence not only the aerodynamic behavior but also the stability of the product, especially during storage and shipment (Wu et al., 2010). The ability to detect, measure, and manipulate these properties can yield an overall more consistent and desirable dry inhalable powder. To this end, researchers have used several techniques to unveil different physicochemical properties of materials, which are summarized in Table 1. As the primary size and morphology of particles affects their aerodynamic size, evaluating these metrics may provide insight into future aerosol performance. The behavior of the powder is then studied by evaluating flow and dispersion properties to assess the potential for efficient aerosolization and effective delivery of an inhaled therapeutic agent.

### 4.1 Physicochemical

Crystallinity is an important property for dry powder analysis. A powder’s crystalline state can influence its stability during storage. Amorphous regions, especially at the particle surface, contribute to interactions and susceptibility to water uptake. Furthermore, the amorphous powder can re-crystallize depending on the relative humidity because water acts as a plasticizer (Shetty et al., 2020). Despite these stability concerns, amorphous particles may also exhibit improved solubility and bioavailability compared to their crystalline counterparts. A common method for assessing crystallinity is X-ray powder diffraction (XRPD). This technique measures the diffraction of X-rays from an atom that structurally repeats and is observed as a sharp peak on the spectra. The amorphous regions lack sharp, thin peaks and are much broader (Hickey, 2018b). While XRPD only assesses the crystallinity of the surface of a particle, the technique remains a valuable tool in the analysis of a powder’s solid-state structure. For example, Maloney et al. (2023d) sprayed dried bedaquiline with the excipient leucine and found that the surface was predominantly amorphous with some degree of crystallinity due to the added excipient leucine.

As previously noted, amorphous regions are prone to water uptake and can affect the long-term stability of dry powders. A technique used to measure water uptake is dynamic vapor sorption. This technique measures a dry powder’s change in mass as a function of relative humidity (Sule and Ambadekar, 2020). Additionally, the moisture content of dry powder formulations can be measured through Karl Fischer (KF) titration and thermogravimetric analysis (TGA) (Hickey, 2018b). In KF titration, a sample is dissolved in a water-free solvent, and iodine is titrated into solution. The titration ends when enough iodine has been added to react with all of the water in solution. With TGA, changes in mass are measured as a function of temperature. Here, a sample is heated, and the mass loss is measured to obtain insights into the water content and thermal stability.

Thermal stability and degradation can be analyzed by differential scanning calorimetry (DSC) and TGA, as previously stated. The DSC technique can be used to measure glass transition temperatures (*T*_g_), melting, crystallization, and degradation (Hickey, 2018b). Typically, amorphous materials have a *T*_g_, which is the temperature at which a material transitions from a ‘glassy’ state to a ‘rubbery’ state. This is important to note because transitioning between states can affect stability during storage and efficacy of the DPI (Shetty et al., 2020).

The molecular interaction between the components of a dry powder can also determine its bioavailability and overall efficacy as a treatment. Fourier transform infrared spectroscopy (FTIR) is a common method for analyzing the bonding between atoms. For example, Paczkowska-Walendowska et al. (2021) confirmed the formation of inclusion complexes between their API and the carrier material, hydroxypropyl-β-cyclodextrin, by observing changes in the absorption spectrum. The molecular composition of a particle’s surface can also be analyzed by X-ray photoelectron spectroscopy (XPS). For example, Stewart et al. (2019) spray dried a spectramide analogue with leucine at an 80:20 mass ratio. Through the use of XPS, the authors reported a decrease in chlorine content on the surface of their powder at a percentage that would indicate that leucine has a preference to accumulate at the surface of the particles.

### 4.2 Primary size

The size distribution of particles in inhalable dry powders can affect aerosol behavior and influence lung penetration. Two common methods for measuring particle size distribution are dynamic light scattering (DLS) and laser diffraction (LD) (Foerter-Barth and Teipel, 2000). DLS measures the size distribution of particles in solution by observing changes in the intensity of the light that scatters due to the Brownian motion of the particles. LD measures changes in the angular diffraction of a laser against a particle that can be in solution or dry. While both techniques measure particle sizing, these methods make assumptions that the particles are spherical and determine the size distribution (intensity or volume distribution) based on the behavior of either its motion in solution or the angular diffraction of a perfectly spherical-shaped particle. Both methods do not account for the actual particle morphology.

### 4.3 Morphology

Particle morphology and shape directly affect the aerodynamic performance of the powder. To observe and image particle morphology, scanning electron microscopy (SEM) and atomic force microscopy (AFM) are used (Hickey et al., 2007a). SEM is commonly employed to image samples in the hundreds of nanometers to micron size range (resolution of 1–10 nm) and to examine the surface and morphology of particles. AFM is a technique that can be used to determine surface structure at the mesoscopic scale (micro-to nanometer). Additionally, AFM can be used as a method to measure surface energy, cohesion and adhesion, and redispersion, and may serve as a tool to predict stability. While SEM is the most commonly used technique to image inhalable dry powders, it lacks resolution for particles in the low-nanometer range. Transmission electron microscopy (TEM) offers an alternative to SEM by generating 2D images of particles at the nanometer scale (resolution of 0.1–1 nm). This technique facilitates the elucidation of the morphology, aggregation of nanoparticles, and inner contents of particles (Almgren et al., 2000).

### 4.4 Behavior

Following physicochemical characterization, the behavior of the dry powder is assessed for its potential efficacy in pulmonary delivery. Simply, the powder behavior can be described through flow and dispersion properties. Current compendial standards focus on the latter, including tests for aerodynamic particle size distribution (APSD) and delivered dose uniformity (DDU). However, these dispersion metrics may have dependence on powder flow, as powders with greater flow and reduced cohesive forces frequently exhibit improved aerosolization.

#### 4.4.1 Flow

Powder flow is often measured and reported using static measurements, including the angle of repose, Carr’s compressibility index (CI), and the Hausner ratio (HR). The angle of repose describes the angle of loosely piled poured powder with respect to horizontal (Hickey et al., 2007b). According to USP General Chapter <1174>, an angle of repose of ≤ 40° indicates a free-flowing powder. Of note, this property is not intrinsic to the powder and varies based on the method used to form the piled powder (USP, 2024). Several studies have reported limited correlation between powder flowability and angle of repose measurements (Hickey et al., 2007b).

Carr’s CI and the HR have been employed as fast and simple methods for empirically estimating powder flow. These methods are described in USP General Chapters <1174> and <616> (USP, 2024, 2025). Carr’s CI is derived from the differences between the tapped and untapped bulk densities and is expressed as a percentage of the tapped bulk density (Carr, 1965; Hickey et al., 2007b). Lower CI values are associated with improved flow. The HR describes a similar metric to the CI as a ratio between the tapped and untapped bulk densities. Aligned with the CI, lower HRs indicate improved powder flow. As mentioned regarding the angle of repose, Carr’s CI and the HR are dependent on the methodology used (e.g., number of times tapped, diameter of cylinder), leading to variability in results across different measurement techniques (USP, 2024). While this method may be valid for understanding the static conditions of bulk powders, flowability under dynamic conditions cannot be suitably determined using such methods because powder flow is determined by both particle properties and external forces (Ogata, 2019).

To further control powder flow assessment, shear cells, such as the Jenike shear cell (Jenike, 1967), were developed. Jenike’s shear cell approach, as an example, considers the effects of bulk powder density, the adhesion and friction between particles, the adhesion and friction between particles and the hopper wall, and the cohesive forces within the powder (Jager et al., 2015). With control over the experimental parameters, the powder flow properties can be determined as a function of various environmental conditions (USP, 2024). There are a range of methods and equipment configurations; thus, a specific methodology is not recommended by USP General Chapter <1174>. However, the variety of equipment highlights the need for a clear description of methodology when presenting data collected using shear cell techniques. Of note, shear cell techniques are useful for evaluating powders that undergo compression but may not be relevant for all aerosol powders (Dunbar et al., 1998).

#### 4.4.2 Dispersion

Compendial standards, such as USP General Chapter <601>, recommend the use of APSD and DDU as dispersion metrics to predict consistent performance of inhaled products (Hickey, 2014; USP, 2012). Although not a direct indicator of lung deposition, pulmonary delivery is influenced primarily by the APSD. It is generally considered that particles with an aerodynamic diameter of <5 μm bypass deposition in the oropharynx and large airways to reach the central and peripheral regions of the lungs (Maloney et al., 2023b). The preferred method for APSD characterization is cascade impaction, where particles released from an inhaler experience changes in the flow direction under laminar flow conditions. Multiple stages are incorporated in a cascade impactor, with each stage collecting smaller particles than the prior stage. Particles below the size threshold of a given stage remain in the flow, whereas particles of greater size impact the surface of the stage (Mitchell et al., 2007). The most common cascade impactors are the 8-stage Andersen cascade impactor (ACI) and the 7-stage next generation impactor (NGI). The NGI has become the most frequently employed device and was developed specifically for sampling pharmaceutical aerosols. Following aerosol sampling, the metrics of mass median aerodynamic diameter (MMAD), geometric standard deviation (GSD), fine particle dose (FPD), and fine particle fraction (FPF) can be calculated to describe the APSD and allow for meaningful comparison between formulations.

DDU is a complementary metric to APSD, highlighting the reproducibility of powder delivery and ultimately lung deposition. A common technique for determining DDU reported in USP General Chapter <601> utilizes a Nephele tube (Hickey, 2014; USP, 2012). Briefly, a Teflon tube of sufficient diameter for a DPI device is sealed, and air flow is drawn through the inhaler and tube to collect powder on the walls of the tube and on a terminal filter. Compendial standards specify that a pressure drop of 4 kPa should be achieved with the inhaler. As inhaler resistances vary, a pressure drop metric is provided rather than a specified flow rate (Maloney et al., 2023b). This technique reduces the analytical errors that may result from calculating the DDU from cascade impaction measurements.

## 5. Therapeutic application

Ultimately, dry powder formulations are intended for pulmonary delivery to a human patient. However, prior to clinical translation, a range of preclinical studies must first be conducted, requiring delivery of dry powder to animals of varying sizes. A range of techniques, devices, and formulations have been used for both preclinical and clinical delivery.

### 5.1 Preclinical

Preclinical dry powder administration can be divided into two main strategies: intratracheal administration and passive inhalation. With intratracheal administration, a tube is inserted into the trachea of an anesthetized animal, and a dry powder is dispersed into the lungs via the airflow from a syringe (Hickey et al., 2024). Commercial options for intratracheal dry powder delivery include the Penn-Century’s Dry Powder Insufflator^™^ (Century, 2013), which is now discontinued, or Aptar Pharma’s Powder Administration Device for Animals (PADA) (Aptar, 2025). Custom-made delivery systems have also been reported (Maloney et al., 2023c; Qiu et al., 2020). Beneficial for initial studies in which powder supply may be low, intratracheal administration requires small powder quantities and delivers the dose directly to the lungs. However, this delivery method is not physiologically relevant, and deposition is likely different from that occurring with inhalation (Hickey et al., 2024).

Alternatively, passive inhalation chambers have been built to deliver dry powder formulations for preclinical studies. For large animals, aerosols may be generated and delivered through a mask. For small animals, nose-only or whole-body exposure chambers are frequently used, where an aerosol cloud is generated within the chamber using a variety of methods for powder dispersal (Hickey et al., 2024). Passive inhalation systems are more physiologically relevant, but the efficiency of delivery is highly dependent on the anatomy and physiology of the animal. Mice, for example, are obligate nose breathers, requiring powder to pass through the nose and throat to reach the lungs (Maloney et al., 2023c). The dose can be estimated, but pharmacokinetic evaluation is necessary to accurately describe the delivered dose. Passive inhalation is a useful technique when powder quantities are not limited and physiological relevance is of high importance.

### 5.2 Clinical

The objective of delivering drug particles to the lungs is to treat, relieve, or prevent disease. Localized diseases can be effectively treated by direct administration to the lungs. The symptoms of local diseases or manifestations of systemic diseases may be relieved by inhalation of aerosols. It is possible to prevent disease with local immunization or immunotherapy.

#### 5.2.1 Non-communicable disease

Various non-communicable diseases can be treated by local drug administration. Dry powder aerosols for the treatment of asthma appeared in the late 1960s with Intal^®^ (disodium cromoglycate delivered from a Spinhaler device; Fisons) (Altounyan and Howell, 2009). Further developments in dry powder technology and drug delivery led to the delivery of Ventolin^®^ (albuterol delivered from a Rotahaler device; Glaxo), Bricanyl^®^ (terbutaline delivered from a Turbuhaler; Astra), and Berotec^®^ (fenoterol delivered by an inhalator device; Boehringer Ingelheim) (Groth and Dirksen, 1983; Lopes Dos Santos et al., 1991). These early products were inefficient but effective for a small portion of the patient population. For decades, dry powder products have been a modest contributor to the overall market for inhaled products. However, in 2003, Advair^®^ (salmeterol/fluticasone delivered by Diskus device, GSK) was approved for chronic controlling therapy of asthma rather than for relief of acute disease (Nelson, 2001). This breakthrough in combination drug therapy delivered through an efficient device elevated dry powder inhaler treatment to a significant component of the overall market. This product has since been superseded by Breo^®^ (vilanterol/fluticasone delivered from the Ellipta device; GSK), which offers the advantage of daily rather than twice daily dosing for disease therapy (Devillier et al., 2018).

Chronic obstructive pulmonary disease (COPD) has many of the same symptoms as asthma but is often accompanied by lung infection with corresponding manifestations of local immunity (Bollmeier and Hartmann, 2020). The bronchoconstriction symptoms of COPD are responsive to anticholinergic drugs. A breakthrough in inhaled therapy occurred in 2004 when Spiriva^®^ (tiotropium delivered from the Handihaler; Boehringer Ingelheim) became available for COPD therapy (Anzueto and Miravitlles, 2020). This long-acting anticholinergic agent is a valuable tool for chronic control of COPD symptoms. Combination therapies, including anti-inflammatories, have come to market and been added to regimens suitable for COPD treatment. Products such as Advair and Symbicort^®^ (formoterol/budesonide delivered by the Turbuhaler; AstraZeneca) were used for COPD, and ultimately, the concept of combining three agents resulted in Trelegy^®^ (vilanterol/fluticasone/umeclidinium delivered from an Ellipta device; GSK) (Mannino et al., 2022).

**Fig. 2:**
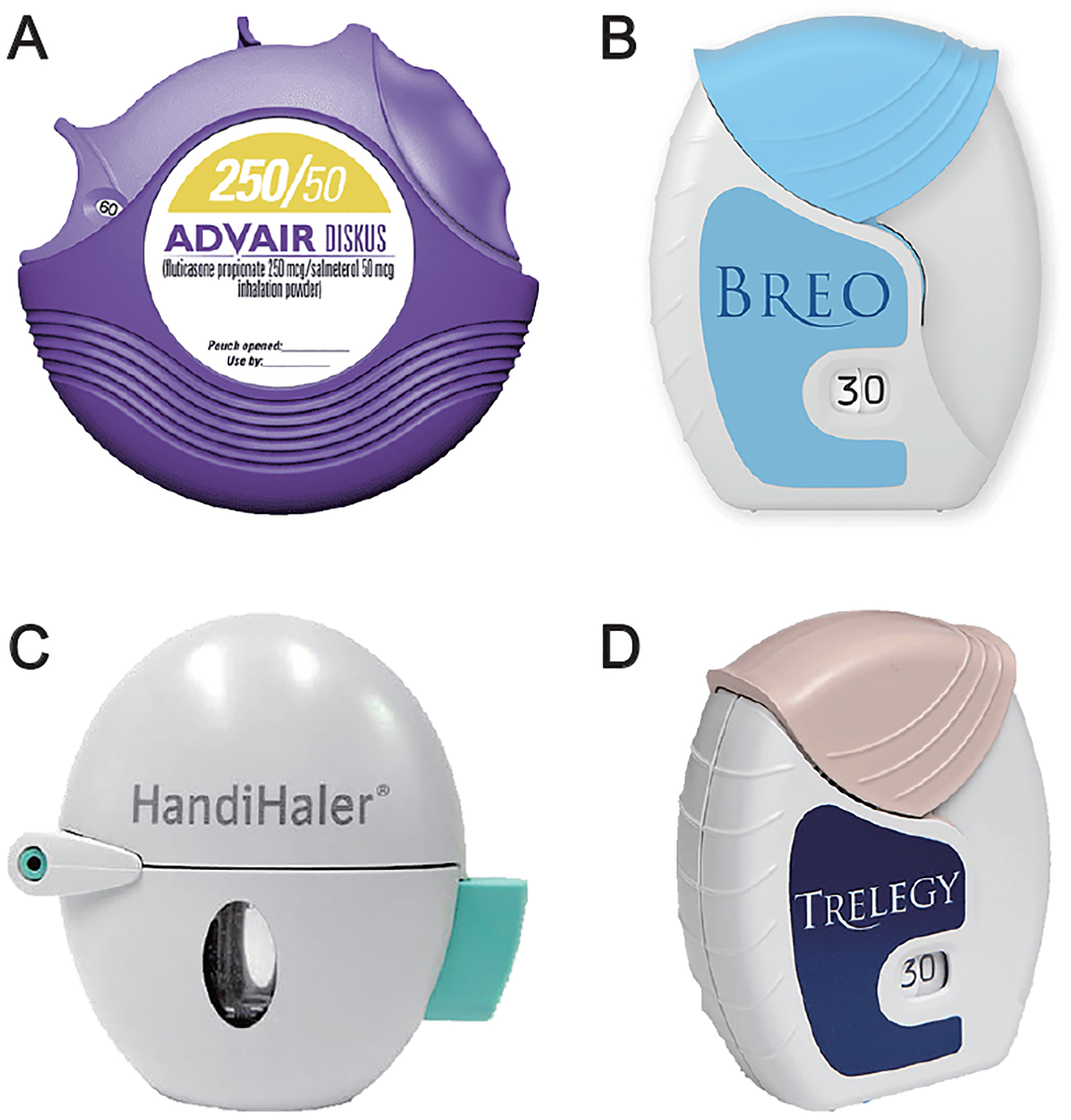
Images of commercial DPI products utilizing lactose blends: **(A)** Advair Diskus^®^ (GSK, 2021), **(B)** Breo^®^ Ellipta^®^ (GSK, 2023a), **(C)** Spiriva^®^ Handihaler^®^ (Boehringer Ingelheim, 2025; FPAN, 2025), and **(D)** Trelegy^™^ Ellipta^®^ (GSK, 2023b).

The formulation of powder in each of these drug products is based on the high potency of each drug, which requires a relatively small dose. Consequently, the two major formulation approaches are carrier lactose blends (Advair, Breo, Trelogy, Spiriva) and controlled aggregation (Symbicort) (Dunbar et al., 1998; Hickey, 2023; Hickey et al., 2007a; Telko and Hickey, 2005). These products are illustrated in Fig. 2.

#### 5.2.2 Communicable disease

**Fig. 3:**
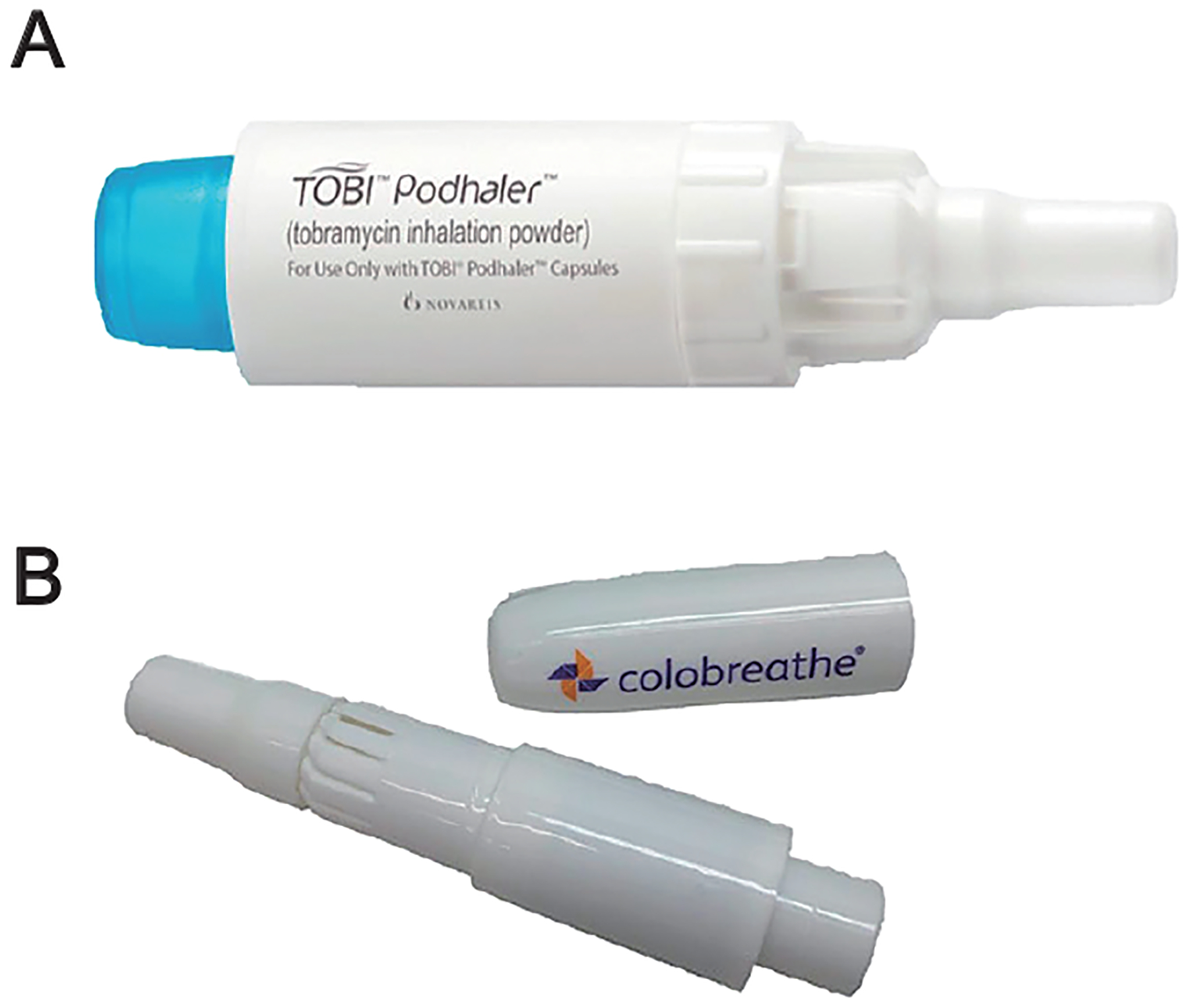
Images of low-potency, high-dose commercial DPI products: **(A)** TOBI^®^ Podhaler^®^ and **(B)** Colobreathe^®^ Turbospin^®^. Reproduced with permission from Ref. (Sibum et al., 2018). Copyright: (2018) Elsevier.

Communicable diseases can be caused by a variety of infectious agents, including prokaryotic organisms (e.g., bacteria and fungi) and obligate intracellular parasites (e.g., viruses). Drugs that are effective in treating infections are often derived from natural sources and are complex antimicrobial agents with low potency and efficacy only at high doses. For most of the past century, aerosols used to treat pulmonary infection were delivered from nebulized solutions, which allowed for an extended delivery time on multiple breaths, resulting in relatively high doses (Hickey and Montgomery, 2004; Miller et al., 1950). However, it should be noted that absorption and clearance rates limit the maximum dose available at any instant in the airways (Suarez and Hickey, 2001). The observation that particles could be manufactured to be readily dispersible in aerodynamic sizes suited for pulmonary delivery, as noted above, created an opportunity for dry particle delivery of antimicrobial agents to treat pulmonary disease. The most prominent commercial example of this approach was the development of TOBI^®^ (tobramycin delivered from the Podhaler; Novartis) for the treatment of *Pseudomonas aeruginosa* infection in cystic fibrosis patients (Fig. 3(A)) (Geller et al., 2011). This was followed by Colobreathe^®^ (colistomethate sodium, delivered by the Turbospin device; Teva/Essential Pharma) (Fig. 3(B)) (Schwarz, 2015). These examples demonstrate the feasibility of developing dry powder products with low-potency, high-dose powder formulations.

**Fig. 4:**
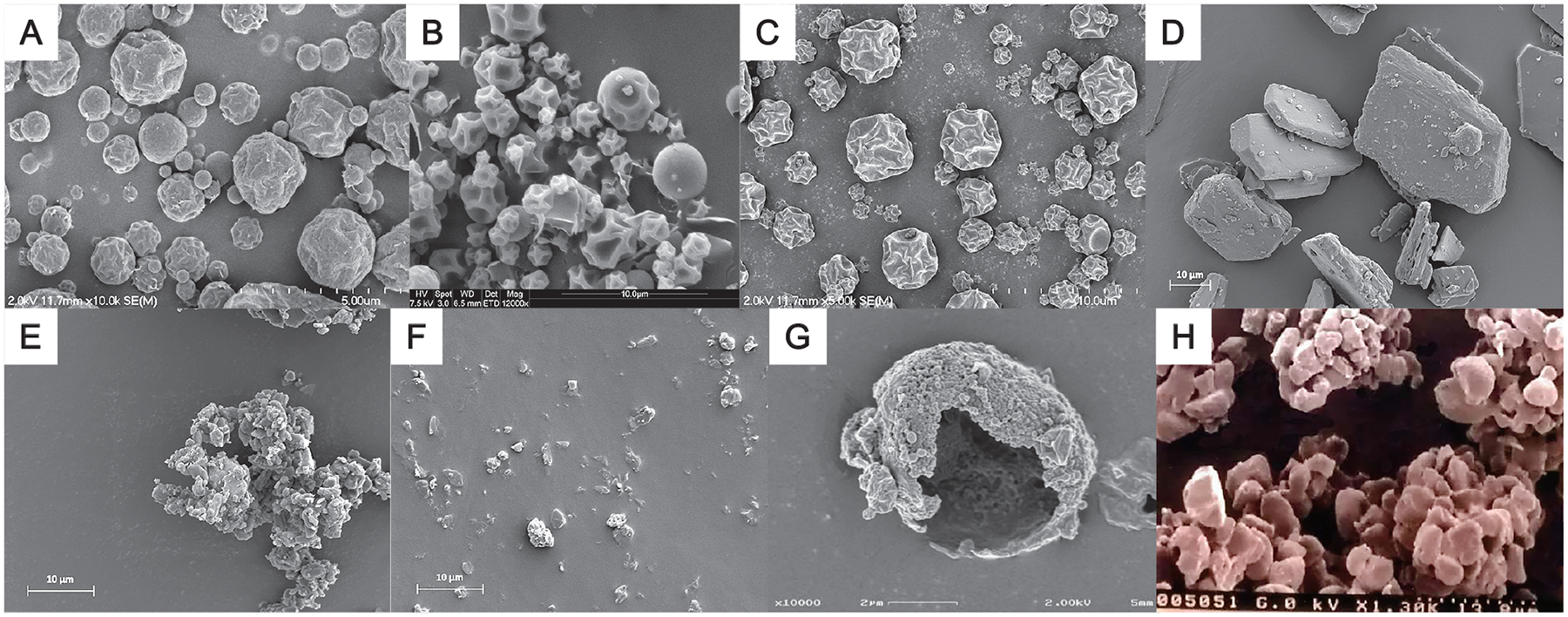
Scanning electron microscopy images of **(A)** spray-dried spectinamide 1599, **(B)** spray-dried CPZEN-45 (reproduced with permission from Ref. (Stewart et al., 2022). Copyright: (2022) Springer), **(C)** spray-dried tigecycline, **(D)** unprocessed clofazimine, **(E)** milled clofazimine prior to dispersion (agglomerated state), **(F)** clofazimine following dispersion from a RODOS dry powder disperser at 3 bar pressure (images **(D)**–**(F)** provided by Dr. Ashlee Brunaugh), **(G)** spray-dried porous microparticles comprised of rifampicin–PLGA nanoparticles (reproduced with permission from Ref. (Sung et al., 2009). Copyright: (2009) Springer), and **(H)** freeze-dried and milled liposome powder containing soy phosphatidylcholine, cholesterol, lactose, and carboxyfluorescein (Schreier et al., 1994).

The ability to deliver high doses offers the potential to deliver new drugs that are not suitable for delivery by other routes of administration or to reuse older drugs for other infectious diseases. Pulmonary mycobacterial infections are frequently the cause of morbidity and, over time, are associated with significant mortality. The most prominent of these is *Mycobacterium tuberculosis*. The global incidence of tuberculosis (TB) is the most significant cause of death from a single microorganism (Braunstein et al., 2019). Several new drugs with no oral bioavailability, which therefore cannot be administered by the ingestion of tablets, have been spray dried and demonstrated efficacy in animal models of diseases when delivered by the pulmonary route (Gonzalez-Juarrero et al., 2021; Stewart et al., 2019; Young et al., 2022). Example images of spectinamide 1599 and CPZEN-45 particles prepared by this method for lung delivery are shown in Fig. 4(A) and (B). Parenteral administration of capreomycin, a second-line agent for multidrug-resistant TB, while therapeutically beneficial, has logistical shortcomings because of the need for an injectable product. Spray drying of capreomycin was shown to be efficacious in a guinea pig model of TB and was well tolerated in a Phase I single escalating dose clinical trial in healthy volunteers (Dharmadhikari et al., 2013; Fiegel et al., 2008). The path to commercial development of these products has been well established (Hickey et al., 2013).

Delivery of drugs as aerosols for TB therapy has been contemplated for at least 75 years, and the field continues to grow rapidly (Miller et al., 1950). Recently, the increasing incidence of non-tuberculous mycobacterial (NTM) infection, notably with *Mycobacterium avium* complex and *Mycobacterium abscessus*, has encouraged a shift in focus for both infectious disease researchers and pharmaceutical scientists to address this unmet medical need. The spray drying of tigecycline is an example of extrapolation from the knowledge gained in treating TB with aerosols (Fig. 4(C)) (Maloney et al., 2023a). Pulmonary delivery of tigecycline through passive inhalation has been shown in a mouse model of disease to be effective in treating *M. abscessus* (Maloney et al., 2023a).

In discussing the clinical application of dry powders consisting of particles suitable for high-dose delivery of low-potency drugs, the barriers that must be overcome to establish an upper limit should be considered. Both particle properties and patient factors require consideration. Clearly, the amount of powder that an individual can inhale in a single breath must be considered in conjunction with the number of metering units (capsules, blisters) corresponding to the number of breaths required to administer the dose. A critical factor at the center of these considerations is the density of the powder and, as a consequence, the volume it occupies. For a given mass, a lower density corresponds to a larger volume, thereby increasing the number of metered doses and inhalations needed to deliver the intended dose. Earlier in the text, it was highlighted that spray drying moved the field forward because it resulted in low-density, easily dispersible particles that could now be generated in high doses. However, these particles occupy a large volume. To increase the dose further but maintain a small number of metered amounts (i.e., breaths), a method is required to lower the density without substantially increasing the volume of the drug particles. This is a difficult but not impossible task.

Spray-dried particles with the desired dispersion and aerodynamic properties are frequently hollow shell or collapsed shell-like particles. Their low apparent density is derived from the amount of air that they encapsulate. However, particles in which the core space is occupied by small particles (nanoparticles) are also low-density but carry greater mass in a unit volume. This observation was developed using nanomilled, microaggregated particles to prepare two drugs for potential aerosol delivery to treat NTMs, clofazimine and tigecycline, as represented in Fig. 4(D)–(F) (Brunaugh et al., 2022; Nair and Smyth, 2023). Notably, these formulations do not require additives, and consequently, the entire mass is drug, which also reduces the required volume to achieve a dose and thereby minimizes the number of metered masses and breaths required for treatment.

In the previous examples in this section, the goal of maximizing the dose to ensure sufficient drug delivery for efficacy in the lungs is based on achieving uniform concentrations that exceed the minimum inhibitory concentration for the microorganism in the airways and lung tissue. This need not be the only therapeutic approach.

Mycobacteria are largely intracellular microorganisms in that they co-opt macrophages, the first line of defense in the immune system of the lungs, for replication and dissemination to other regions of the lungs and systemically (Mortensen and Hickey, 2014). Consequently, relatively small amounts of drug can be incorporated into polymeric particles that target macrophages and deliver locally within the cell, facilitating high doses in the immediate vicinity of the infecting mycobacteria and treating the disease. Targeting strategies emphasize locally high doses, achieved with small quantities of drug, rather than globally high doses, achieved with large quantities of drug. The emphasis on keeping volumes low is less important in this scenario because large powder doses are not required. This principle was demonstrated using PLGA particles initially formulated as solid microparticles administered from a carrier lactose blend and subsequently as nanoparticles forming the shell in hollow spray-dried microparticles (Fig. 4(G)) (O’Hara and Hickey, 2000; Sung et al., 2009). Both preparations demonstrated efficacy in a guinea pig model of TB (Garcia Contreras et al., 2015; Suarez et al., 2001a; b).

## 6. Future prospects

Dry particle structure and manufacture have evolved significantly over the last two decades. Considering likely future developments, particle technology will continue to evolve, especially with respect to composition. This is best illustrated by considering general trends in new drug development and the prospects that these advances will contribute to further developments in the field.

New drug approvals from the US FDA are increasing in the category of biological agents (Martins et al., 2022). Broadly speaking, these may be considered macromolecules, notably proteins, polypeptides, and nucleic acids. In the 1990s, the biotechnology industry faced the challenge of finding alternative routes of administration for therapeutic agents that could only be administered by injection. The appearance of Dornase^™^ (DNase; Genentech), the first approved protein for inhalation, used to treat CF, created the opportunity for the rapid development of other candidate biological agents for delivery via this route (Gonda, 1996).

Dornase^™^ is delivered by nebulization, but as other molecules were being considered, dry powder products rapidly became the desired dosage form. In this arena, the focus on insulin as a candidate for inhalation therapy was responsible for an enormous effort in research and development. The result of this work was two approved products: Exubera^™^ (Pfizer/Nektar) and Afrezza^®^ (Mannkind) (Patton, 2005; Setji et al., 2016). The exciting developments in this arena, such as spray-dried particle manufacture and protein delivery, overshadowed equally important developments in additives either adopted or created to facilitate the success of these products. Phospholipids, amino acids, and stabilizing sugars became part of spray drying options (Edwards et al., 1997; Geller et al., 2011; Patton, 2005; Vehring, 2008). In addition, fumaryl-diketopiperazine, a new excipient, has emerged as a valuable component of particles for pulmonary delivery (Setji et al., 2016). These developments may be of importance as interest in delivering therapeutic antibodies increases. During the COVID-19 pandemic, experiments were conducted to demonstrate the benefit of antibodies that bind to the virus (SARS-CoV-2) or its cellular binding receptor (ACE-2) to prevent infection (Datta et al., 2020; Farrell et al., 2022; Ge et al., 2021). Local delivery of mucotrapping antibodies via aerosol administration confirmed that direct delivery to the lungs is a viable approach to therapy (McSweeney et al., 2023). It can also be expected that immunotherapeutic antibodies used in cancer treatment could be delivered directly to the lungs to treat local diseases.

Pulmonary delivery of nucleic acid therapeutics began 30 years ago with the administration of DNA plasmids to correct the genetic deficiency in chloride ion transport that occurs in CF (Stribling et al., 1992). Early dry powder formulations were prepared by freeze-drying and milling the plasmid in a lactose matrix (Fig. 4(H)) (Holzner et al., 1998; Schreier et al., 1994). Transfection using a luciferase marker was demonstrated in an in vitro physiologically relevant cell model with this formulation. Initial interest in the use of RNA to intervene in gene expression focused on the use of siRNA and oligonucleotides (Paul et al., 2023; Zimmermann et al., 2022). The success of mRNA delivery in solid lipid nanoparticles opens the potential to use translational or transcriptional methods to modulate gene expression in the lungs (Gordon et al., 2024; Liu et al., 2024; Sarode et al., 2024). As indicated earlier, the possibility of preparing spray-dried microparticle/nanoparticle-shell systems is ideal for the delivery of these therapeutic moieties.

## 7. Conclusions

Crucial advances in manufacture, characterization, and preclinical assessment have supported the development of new therapies for a variety of non-contagious and contagious diseases. The currently available options may support a new generation of inhaled therapies suitable for more targeted biological activity.

## Figures and Tables

**Table 1:** Techniques for the physicochemical characterization of dry powder formulations.

Analysis	Technique	Purpose
Physicochemical	X-ray powder diffraction (XRPD)	Identify surface crystal structure, phase purity, and crystallinity
	Fourier transform infrared spectroscopy (FTIR)	Identify interactions between the drug, excipient, and carrier system
	X-ray photoelectron spectroscopy (XPS)	Measure atomic composition of the surface of particles
	Differential scanning calorimetry (DSC)	Measure glass transition temperature (*T*_g_), melting point, and crystallinity
	Thermogravimetric analysis (TGA)	Assess thermal stability and moisture content
	Karl Fischer (KF)	Measure moisture content
	Dynamic vapor sorption (DVS)	Assess moisture absorption
Primary size	Dynamic light scattering (DLS)	Determine the size distribution profile of particles in solution
	Laser diffraction (LD)	Determine the size distribution of particles
Morphology	Scanning electron microscopy (SEM)	Image the 3D surface structure of particles
	Atomic force microscopy (AFM)	Image and examine surface structure and topography
	Transmission electron microscopy (TEM)	Image particles in 2D down to nanometers in size

## Data Availability

Data are available upon request from the authors.

## Nomenclature

2D: two-dimensional
3D: three-dimensional
ACI: Andersen cascade impactor
AFM: atomic force microscopy
API: active pharmaceutical ingredient
APSD: aerodynamic particle size distribution
CF: cystic fibrosis
CI: compressibility index
COPD: chronic obstructive pulmonary disease
DDU: delivered dose uniformity
DLS: dynamic light scattering
DNA: deoxyribonucleic acid
DPI: dry powder inhaler
DSC: differential scanning calorimetry
DVS: dynamic vapor sorption
FDA: Food and Drug Administration
FPD: fine particle dose
FPF: fine particle fraction
FTIR: Fourier transform infrared spectroscopy
GSD: geometric standard deviation
HR: Hausner ratio
KF: Karl Fischer
LD: laser diffraction
MDI: metered dose inhaler
MMAD: mass median aerodynamic diameter
mRNA: messenger ribonucleic acid
NGI: next-generation impactor
NTM: nontuberculous mycobacteria
PLGA: poly(lactic-co-glycolic acid)
RNA: ribonucleic acid
SCF: supercritical fluid
SEM: scanning electron microscopy
siRNA: small interfering ribonucleic acid
TB: tuberculosis
TEM: transmission electron microscopy
TFF: thin film freezing
*T*_g_: glass transition temperature
TGA: thermogravimetric analysis
USP: United States Pharmacopeia
XPS: X-ray photoelectron spectroscopy
XRPD: X-ray powder diffraction
